# *SERPINA1* methylation and lung function in tobacco-smoke exposed European children and adults: a meta-analysis of ALEC population-based cohorts

**DOI:** 10.1186/s12931-018-0850-8

**Published:** 2018-08-22

**Authors:** Anna Beckmeyer-Borowko, Medea Imboden, Faisal I. Rezwan, Matthias Wielscher, Andre F. S. Amaral, Ayoung Jeong, Emmanuel Schaffner, Juha Auvinen, Sylvain Sebert, Ville Karhunen, Robert Bettschart, Alexander Turk, Marco Pons, Daiana Stolz, Florian Kronenberg, Ryan Arathimos, Gemma C. Sharp, Caroline Relton, Alexander J. Henderson, Marjo-Riitta Jarvelin, Deborah Jarvis, John W. Holloway, Nicole M. Probst-Hensch

**Affiliations:** 1 Swiss Tropical and Public Health Institute, Socinstrasse 57, 4002 Basel, Switzerland; 2 University of Basel, Basel, Switzerland; 3 Human Development and Health, Faculty of Medicine, University of Southampton, Southampton, UK; 4 Department of Epidemiology and Biostatistics, MRC-PHE Centre for Environment and Health, School of Public Health, Imperial College London, W2 1PG, London, UK; 5 Population Health and Occupational Disease, NHLI, Imperial College London, London, UK; 6 Center for Life Course Health Research, University of Oulu, Oulu, Finland; 7 Oulunkaari Health Center, Ii, Finland; 8 Medical Research Center, University Hospital of Oulu, University of Oulu, Oulu, Finland; 9 Biocenter Oulu, University of Oulu, Oulu, Finland; 10 Department for Genomics of Common Diseases, School of Public Health, Imperial College London, London, UK; 11 Lungenpraxis Aarau, Hirslanden Klinik, Aarau, Switzerland; 12 Zürcher Höhenklinik, Wald, Switzerland; 13 Ospedale Regionale di Lugano-Sede Civico, Lugano, Switzerland; 14 Clinic of Pulmonary Medicine and Respiratory Cell Research, Basel, Switzerland; 15 Division of Genetic Epidemiology, Department of Medical Genetics, Molecular and Clinical Pharmacology, Innsbruck Medical University, Innsbruck, Austria; 16 MRC Integrative Epidemiology Unit, University of Bristol, Bristol, UK; 17 Department of Population Health Sciences, Bristol Medical School, University of Bristol, Bristol, UK; 18 Bristol Dental School, University of Bristol, Bristol, UK; 19 Unit of Primary Health Care, Oulu University Hospital, OYS, Kajaanintie 50, 90220 Oulu, Finland; 20 Department of Life Sciences, College of Health and Life Sciences, Brunel University London, Kingston Lane, Uxbridge, Middlesex UB8 3PH UK

**Keywords:** Chronic obstructive pulmonary disease; *SERPINA1* methylation, DNA methylation, Epigenetics, Population based study, Smoking, Alpha-1 antitrypsin

## Abstract

**Background:**

The pathophysiological role of *SERPINA1* in respiratory health may be more strongly determined by the regulation of its expression than by common genetic variants. A family based study of predominantly smoking adults found methylation at two Cytosine-phosphate-Guanine sites (CpGs) in *SERPINA1* gene to be associated with chronic obstructive pulmonary disease risk. The objective of this study was to confirm the association of lung function with *SERPINA1* methylation in general population samples by testing a comprehensive set of CpGs in the *SERPINA* gene cluster. We considered lung function level and decline in adult smokers from three European population-based cohorts and lung function level and growth in tobacco-smoke exposed children from a birth cohort.

**Methods:**

DNA methylation using Illumina Infinium Human Methylation 450 k and EPIC beadchips and lung function were measured at two time points in 1076 SAPALDIA, ECRHS and NFBC adult cohort participants and 259 ALSPAC children. Associations of methylation at 119 CpG sites in the *SERPINA* gene cluster (*PP4R4-SERPINA13P*) with lung functions and circulating alpha-1-antitripsin (AAT) were assessed using multivariable cross-sectional and longitudinal regression models.

**Results:**

Methylation at cg08257009 in the *SERPINA* gene cluster, located 32 kb downstream of *SERPINA1*, not annotated to a gene, was associated with FEV_1_/FVC at the Bonferroni corrected level in adults, but not in children. None of the methylation signals in the *SERPINA1* gene showed associations with lung function after correcting for multiple testing.

**Conclusions:**

The results do not support a role of *SERPINA1* gene methylation as determinant of lung function across the life course in the tobacco smoke exposed general population exposed.

**Electronic supplementary material:**

The online version of this article (10.1186/s12931-018-0850-8) contains supplementary material, which is available to authorized users.

## Background

Tobacco smoke represents the most important COPD risk factor. Yet, not all smokers develop the disease during their lifetime [1]. The interaction between rare genetically determined alpha-1 antitrypsin (AAT) deficiencies and smoking on emphysema risk illustrates the relevance of genetic susceptibility and gene-environment interactions. The protease inhibitor AAT, encoded by *SERPINA1*, prevents the extracellular matrix degradation and destruction of alveoli by neutrophil. Both, tobacco smoke and AAT deficiency cause neutrophil recruitment in the lung. Pro-inflammatory and oxidative processes further diminish the anti-proteolytic AAT activity [2, 3].

Genome-wide association studies (GWAS) initially suggested that common *SERPINA1* variants might influence COPD risk and associated lung function phenotypes. However, reported associations with common *SERPINA1* single nucleotide polymorphisms (SNPs) were shown to reflect their linkage with more penetrant rare variants [4, 5]. In a recent GWAS, restricted to smokers, *SERPINA1* variants were not associated with the level of lung function [6]. In the largest GWAS meta-analysis to date, a genetic risk score including 95SNPs, independently associated with lung function or COPD, did not contain SNPs from *SERPINA1*. Nevertheless, an over-representation of genetic variants related to elastic-fibre pathway was observed [7].

*SERPINA1* gene is complex with eleven splicing isoforms differing in the *SERPINA1* 5’-UTR, tissue expression and secondary structure. This suggests that the gene’s pathophysiological role may be more strongly determined by differences in expression, regulation or posttranscriptional modification than by genetic variation [8]. A recent family-based study of predominantly smoking adults without severe AAT deficiency investigated *SERPINA1* methylation. It was associated with COPD risk, forced expiratory volume in 1 s (FEV_1_) and the ratio of FEV_1_ to forced vital capacity (FEV_1_/FVC) [9] at two CpG sites.

Our candidate gene study is the first to investigate the influence of *SERPINA1* methylation on lung function in a general population. Specifically, we tested the association of a comprehensive set of methylation signals in the *SERPINA* gene cluster with lung function levels; with 10 to 15-year lung function decline in adult smokers from three population-based European cohorts; and with lung function growth in tobacco-smoke exposed children from the ALSPAC birth cohort.

## Methods

### Study design

Cross-sectional and longitudinal analyses used data from three European adult cohorts and one European birth cohort with longitudinal data on lung function and DNA methylation (DNAm).

### Ethics approval and consent to participate

All studies were approved by the local ethics committees and participants or their guardians provided written consent prior to taking part in the study.

### Adult cohorts and participants

Participants came from three population-based studies: Swiss Study on Air Pollution and Lung and Heart Disease in Adults (SAPALDIA) [10, 11], European Community Respiratory Health Survey (ECRHS) [12] and Northern Finland Birth Cohort 1966 [13]. Participation across the studies included structured questionnaires, pre-bronchodilation spirometry, and blood sampling for DNA extraction and analysis. SAPALDIA and ECRHS share a harmonized respiratory health protocol. The study sample was restricted to ever smokers, aged ≥25 years, with data on valid lung function, relevant covariates, and DNA samples from two follow-ups subjected to methylome typing in the context of the Aging Lungs in European Cohorts (ALEC) project. The final sample size was 1076 (*n* = 561 SAPALDIA, *n* = 267 ECRHS, and *n* = 248 NFBC).

### Children and adolescent cohort and participants

The Avon Longitudinal Study of Parents and Children (ALSPAC) consisted of 68 follow-up assessments between birth and 18 years [14]. Spirometry was performed at ages 8.5 and 15 years. Participants were restricted to children and adolescents with DNA methylome and valid lung function data from two time points, as well as information on relevant covariates. The study sample included 259 children exposed to tobacco-smoke (mother smoked during pregnancy and/or lived with a smoker and/or reported smoking ≥twice in their lifetime).

### Lung function

Pre-bronchodilation spirometry was performed by trained personnel according to the ATS/ERS recommendations [15]. FEV_1_, FVC, and FEV_1_/FVC were the lung function parameters considered. In SAPALDIA, parameters were derived from 2001 and 2010 measurements and corrected for change in spirometers from SensorMedics to ndd EasyOne [16] in ERCHS they were derived from 1998 and 2008 measurements and were corrected from several spirometers to ndd EasyOne. In NFBC they were measures by Vitalograph P-model in 1997 and MasterScreen Pneumo spirometer in 2012. ALSPAC parameters were derived from 2000 and 2016 spirometries obtained from the same brand spirometer [17].

### DNA methylation measurement

DNAm of autosomes obtained at two time points of lung function measurements was the predictor of interest. DNA was extracted from peripheral whole blood in all cohorts. In SAPALDIA and ALSPAC, DNAm was measured at both time points using the Infinium HumanMethylation450K BeadChip (Illumina, Inc.), in NFBC using the Infinium HumanMethylation450K BeadChip at the first time point and the EPIC BeadChip at the second time point and in ECRHS, at both time points using the EPIC BeadChip.

For ECRHS and SAPALDIA, randomized distribution of samples for bisulphite conversion was applied and for methylome typing batches, the samples from each time point from the same person were placed next to each other on the array. In NFBC1966 DNAm data were recorded in two batches following the clinical assessments of participants aged 31 and 46.

The methylation level (β value) was derived from raw intensities after pre-processing using R package using *minfi* [18] followed by beta-mixture quantile normalization (BMIQ) [19] in SAPALDIA or *RnBeads* [20] followed by quantile normalization (QN) in ECRHS. In NFBC, CPACOR pipeline [21] was used to pre-process and prepare β values. In all adult cohorts, normalized beta scores were regressed on principle components derived from the array control probes reflecting technical bias. The resulting residuals were used as predictors in the associations with lung function.

In ALSPAC, analogous standard quality control procedures were applied, in addition, genotype probes on the HumanMethylation450K were compared between samples from the same individual and against SNP-chip data to identify and remove any sample mismatches. Data were pre-processed in R (version 3.0.1) with the WateRmelon package according to the subset quantile normalization approach [22] to reduce the non-biological differences between probes. Technical batch effect for each methylation time-point was adjusted for by including ten surrogate variables into the models.

### Methylation signals considered in *SERPINA* gene cluster

The human serine protease inhibitor (serpin) gene cluster is located at 14q32. It consists of eleven functionally diverse serpin genes within a region of approximately 400 kb in length. Gene sub-clusters consist of four, three, and four genes each. The best characterized and proximal sub-cluster with a length of about 107 kb includes *SERPINA1* as well as an antitrypsin-related pseudogene (*ATR*, *SERPINA2;*∼13 kb downstream), the corticosteroid-binding globulin gene (*CBG*, *SERPINA6;*∼68 kb downstream), and the protein Z inhibitor gene (*ZPI*, *SERPINA10;*∼100 kb downstream).

**Fig. 1:**
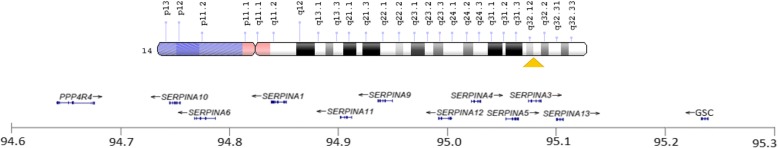
Chromosome 14 and *SERPINA* gene cluster located between *PPP4R4* and *GSC* genes

**Fig. 2:**
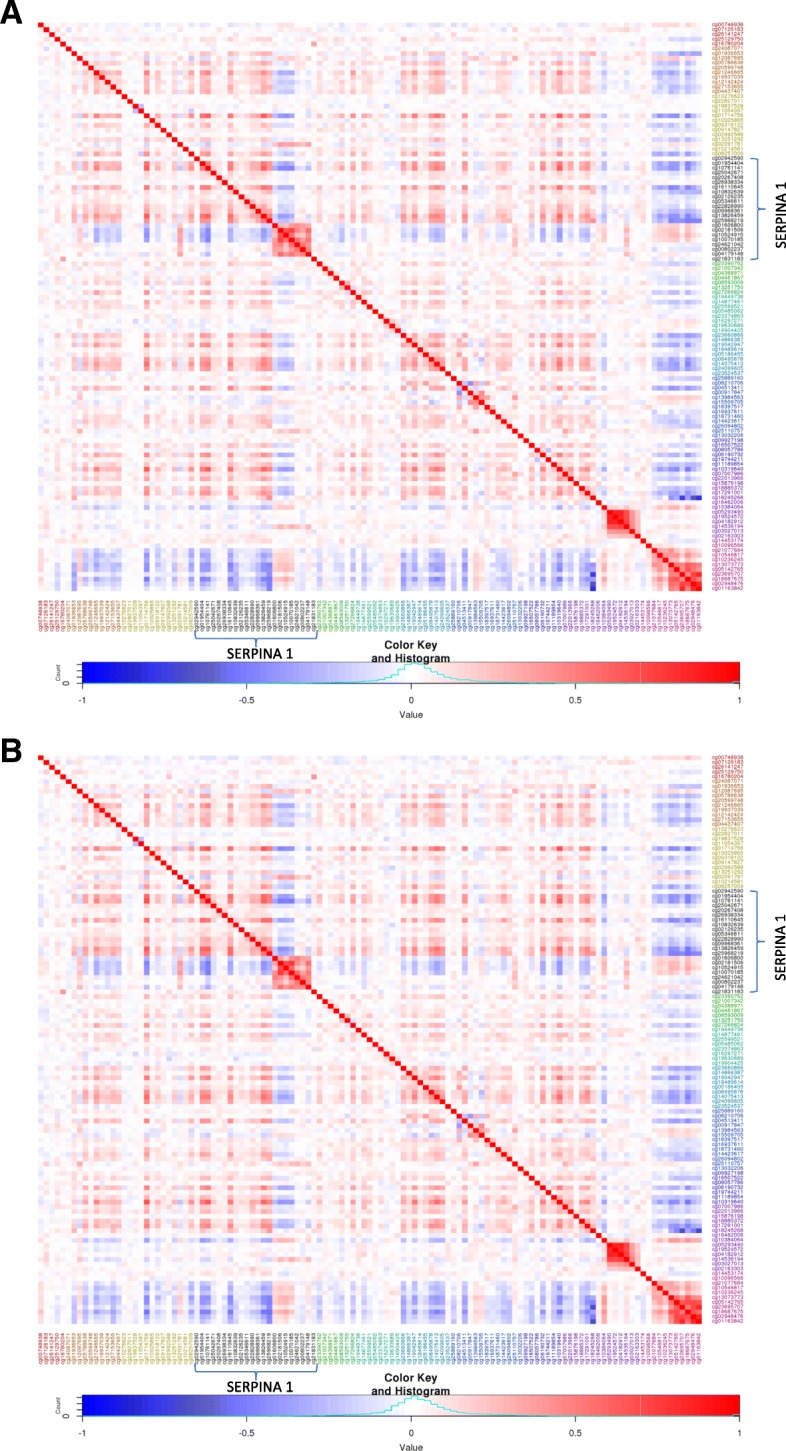
**a-b** Heatmaps for correlation of methylation at 119 CpGs in *SERPINA* gene cluster in SAPALDIA. Heatmaps correlations at the first (**a**; T1) and second (**b**; T2) time-points. CpGs located on the *SERPINA1* gene are highlighted in black and labeled. Both figures were created with the R software

Because the tissue-specific expression of different genes in the serpin cluster is regulated by chromosomal elements and chromatin structure, and in the absence of knowledge about the relevance of more distal methylation signals on gene expression, we included in the analysis all 119 CpGs located 99 kb downstream (*PPP4R4*) and 376 kb upstream (*GSC*) from the *SERPINA1* gene. The CpGs were allocated to 12 genes: *PPP4R4, SERPINA10, SERPINA6, SERPINA1, SERPINA11, SERPINA9, SERPINA12, SERPINA4, SERPINA5, SERPINA3, SERPINA13*, and *GSC* (Fig. 1: location of CpG sites considered; Fig. 2: correlation of methylation at different CpGs at both time points).

### Covariate information

Information on participant’s age, sex, education, height, and smoking status was derived from questionnaires administered during the two time-points. Cell proportions in the respective blood samples were estimated using the Houseman method [23] implemented in the minfi package [18].

For sensitivity analysis, SAPALDIA provided information on concentrations of high-sensitive C-reactive protein (CRP) and AAT in blood samples collected at the first time point. CRP and AAT concentrations were measured by latex-enhanced immunoturbidimetric assays (Roche Cobas Integra analyzer; Roche Diagnostics). Inter-assay coefficients of variation were below 5% and lower detection rate were 0.1 mg/l (CRP) and 0.21 (AAT) [24]. In addition, SAPALDIA provided genetic information on rare AAT deficiency variants PiZ and PiS [4].

### Statistical analyses

#### Cohort-specific analyses on lung function and its decline in adults

Within each adult cohort, four statistical models were run: a) cross-sectional associations of DNAm with lung function at T1, b) cross-sectional associations of DNAm with lung function at T2, c) repeat cross-sectional associations of DNAm and lung function at both time points, and d) predictive associations of DNAm at T1 on annual lung function decline between T1 and T2, calculated as (lung function at T2 - lung function at T1 divided by the time of follow-up in years).

The absolute levels of lung functions (FEV_1_, FVC, FEV_1_/FVC) were regressed on residuals of methylation by fitting linear regression models and a mixed-model with a random effect for the subject (repeat cross-sectional model), respectively. Sample size was kept constant across all models and analyses. Models were a priori adjusted for the following covariates associated with lung function level at *P-value* < 0.05: study center, age, age^2^, education, height, (height-mean(height))^2^, sex, sex*age, sex*age^2^, sex*height, sex*(height-mean(height))^2^, and cell composition (CD8T; CD4T; NK; Bcell; Mono and Eos).

#### Meta-analysis of adult cohort results

The fixed-effect meta-analysis weighted by the inverse of variance was completed using METAL on the 119CpG sites common to all three cohorts [25]. Associations with *P*-values< 0.05 were considered as nominally significant. Since methylation of CpGs in the *SERPINA* cluster did not correlate throughout the whole genetic region (Fig. 2), the total number of CpGs was used as correction for multiple testing. A Bonferroni corrected *P*-value< 4.2 × 10^− 4^ was considered statistically significant. Given the generally medium-to-high correlation between lung function parameters, associations of CpGs with different lung function parameters were not considered independent [7].

#### Cohort-specific analysis on lung function and its growth in children and adolescents

Four regression models equivalent to those assessed in adult cohorts were run to investigate cross-sectional and predictive associations of methylation at 119 CpGs in the *SERPINA* gene cluster with the absolute level and increase in FEV_1_, FVC, and FEV_1_/FVC in children and adolescents. The models were a priori adjusted for covariates associated with lung function at *P*-value< 0.05: study center, age, mother’s education, height, (height-mean(height))^2^, sex, sex*age, sex*height, and estimated cell composition (CD8T; CD4T; NK; Bcell; Mono; Eos) [23].

#### Sensitivity analyses

Seven sensitivity analyses were conducted in SAPALDIA:(1) 119 CpGs from T1 were regressed on circulating AAT measured at T1 [24] by including the same set of covariates as in cross-sectional models on lung function at T1;(2) cross-sectional models at T1 were additionally adjusted for CRP and AAT;(3) for all phenotypes, all four statistical models were additionally adjusted for the presence of PiS and PiZ: 0, 1 or 2 alleles;(4) for neutrophils;(5) stratified by gender;(6) stratified by obesity (BMI 30 < vs. ≥ 30 kg/m^2^;(7) and stratified by self-reported asthma (“Have you ever had asthma?”).

## Results

### Characteristics of adult cohort participants

Characteristics of the adult participants at the first time point (T1) and second time point (T2) are presented in Table 1. SAPALDIA participants were oldest, reported the highest number of pack-years, had lung function levels similar to ECHRS, but lower than NFBC, and exhibited the highest prevalence of COPD based on pre-bronchodilation spirometry and defined as FEV_1_/FVC below the lower limit of normal: in SAPALDIA 15.3% (T1) and 17.1% (T2), respectively, compared to 10.5% (T1) and 12.7% (T2) in ECHRS and 2.2% (T1) and 11.4% (T2) in NFBC. The prevalence of self-reported doctor’s diagnosed asthma was between 10 and 20%, increasing with aging in all three cohorts.

**Table 1:** Population characteristics of adult cohorts

		SAPALDIA2 T1, 2001	SAPALDIA3 T2, 2010	ECRHS 2 T1, 1998	ECRHS 3 T2, 2008	NFBC 1966 T1, 1997	NFBC 1966 T2, 2012
N		561	561	267	267	248	248
Female, %		48.3	48.3	52.1	52.1	49.8	48.2
Age (years), mean (SD)		50.4 (10.8)	58.7 (10.7)	44.0 (6.0)	55.0 (6.0)	31.0 (0.3)	46.3 (0.4)
Height (cm), mean (SD)		170.2 (9.2)	169.5 (9.3)	170.0 (9.0)	170.0 (9.0)	172.0 (8.7)	172.0 (8.6)
Weight (kg), mean (SD)		73.9 (14.8)	75.6 (15.6)	74 (15.0)	77 (16.0)	72.1 (12.8)	80.8 (15.3)
Body mass index (kg/m^2^), mean (SD)		25.4 (4.2)	26.2 (4.6)	25.2 (4.2)	26.8 (4.5)	24.3 (3.6)	27.2 (4.5)
Smoking status, %							
Ex-smoker		52.0	63.0	54.7	69.3	46.8	69.2
Current smoker		48.0	37.0	45.3	30.7	53.2	30.8
Pack-years, mean (SD)^a^		20.4 (20.2)	22.6 (22.1)	17 (17.0)	20 (21.0)	7.7 (5.9)	11.0 (9.6)
Education (%)							
SAPALDIA	ECRHS & NFBC						
Low^b^	≤16 years^c^	5.0	4.0	10.9	10.9	0.4	0.4
Intermediate	17–19 years	66.0	67.0	30.5	30.5	61.9	61.9
High	20+ years	29.0	29.0	58.6	58.6	37.7	37.7
FVC (L), mean (SD)		4.4 (1.0)	4.1 (1.0)	4.4 (1.0)	4.0 (1.0)	4.9 (1.0)	4.6 (0.9)
FVC % Predicted^d^, mean (SD)		106.2 (13.4)	106.0 (13.4)	100.7 (13.0)	99.2 (13.5)	101.8 (10.8)	104.1 (11.2)
FEV_1_ (L), mean (SD)		3.3 (0.8)	3.0 (0.8)	3.4 (0.8)	3.0 (0.7)	4.1 (0.8)	3.5(0.7)
FEV_1_% Predicted^d^, mean (SD)		99.3 (14.2)	97.9 (16.0)	96.5 (13.7)	93.8 (14.2)	102.1 (11.5)	100.1 (11.8)
FEV_1_/FVC, mean (SD)		0.8 (0.08)	0.7 (0.08)	0.8 (0.06)	0.8 (0.1)	0.8 (0.05)	0.8 (0.06)
FEV_1_/FVC % Predicted^d^, mean (SD)		93.2 (9.4)	92.0 (9.9)	95.4 (7.7)	94.2 (7.7)	99.9 (6.8)	95.8 (7.0)
CRP (mg/L), mean (SD)		1.92 (2.8)	–	–	–	–	–
AAT (g/L), mean (SD)		1.27 (0.2)	–	–	–	–	–
COPD, defined by FEV_1_/FVC < LLN^e^		15.3	17.1	10.5	12.7	2.2	11.4
Doctor-diagnosed asthma, %		15.7	19.1	14.6	17.2	10.5	17.1
Respiratory medication (last 12 months), %		22.5	25.8	12.4	14.3	NA	NA
Spirometer brand and model, %							
SensorMedics 2200		100.0	–	–	–	–	–
EasyOne NDD Medical Technologies		–	100.0	–	100.0	–	–
SensorMedics 2400		–	–	36.0	–	–	–
Jaeger Pneumotach		–	–	16.1	–	–	–
Biomedin Spirometer		–	–	47.9	–	–	–
Vitalograph P Model		–	–	–	–	100.0	–
MasterScreen Pneumo		–	–	–	–	–	100.0

Characteristics of ever smokers at first (T1) and second (T2) time points in adult cohorts: SAPALDIA, ECRHS and NFBC 1966

*SD* standard deviation

^a^Excluding lifetime non-smokers

^b^Education: low corresponding to primary education; intermediate corresponding to secondary, middle, or vocational school; and high corresponding to technical college or University

^c^Age at end of studies

^d^Percentage of the predicted value estimated using GLI equations

^e^LLN, lower limit of normal estimated using GLI2012 reference equations

### Association of methylation at 119 CpGs in the *SERPINA1* cluster with lung function in adult ever smokers

#### SERPINA1

Of the 119 CpGs, 17 were located in the *SERPINA1* gene. DNAm at these 17 sites was not associated with any of the three lung function parameters at a Bonferroni-corrected *P*-value, irrespective of the model considered (Tables 2 and 3; Additional file 1: Table ES1). However, for FEV_1_, meta-analysis revealed three nominally significant associations at T1 (cg09968361, cg25968219, cg04179148) (Table 2). The positive association of cg09968361 with levels of FEV_1_, nominally significant at T1 (*β*:1.43, *P*-value = 0.04) was consistent in direction for T2 and the repeat cross-sectional analysis (*β*:0.72, *P*-value = 0.02). However for change in lung function, an increase in methylation at this CpG site was associated with accelerated FEV_1_ decline (*β*:-0.10, *P*-value = 0.011). For FEV_1_/FVC (Table 3), meta-analysis also revealed three nominally significant associations at T1 (cg25968219, cg24621042, cg04179148), which in part overlapped with those associated with FEV_1_, also in terms of direction of association. No association of methylation at *SERPINA1* CpGs and FVC was observed (Additional file 1: Table ES1).

**Table 2:** Results from Meta-Analyses, FEV_1_ and AAT, Adult Cohorts

CpG	CpG Position	Gene Group	SAPALDIA		Meta- Analysis of Lung Function Association in SAPALDIA, NFBC and ECRHS											
			Circulating AAT		Cross-Sectional T1			Cross-Sectional T2			Repeat Cross-Sectional			Change		
			Coef.	*P*.value^a^	Coef.	*P*.value^a^	Direction^b^	Coef.	*P*.value^a^	Direction^b^	Coef.	*P*.value^a^	Direction^b^	Coef.	*P*.value^a^	Direction^b^
cg10761141	94,844,776	3UTR	−0.30	0.25	0.75	0.13	+++	0.63	0.21	+ − +	−0.04	0.84	+ − –	0.02	0.48	+++
cg25042671	94,849,061	Body	**−0.59**	**0.04**	−0.72	0.25	− + −	− 0.80	0.21	– − −	−0.22	0.52	++−	−0.03	0.44	− + −
cg20267408	94,849,703	5UTR	−0.44	0.21	−0.15	0.82	++−	−0.05	0.94	++−	0.10	0.79	++−	0.00	0.97	+ − –
cg26938334	94,849,747	5UTR	1.16	0.42	− 0.01	0.99	− + −	− 0.35	0.74	− + −	− 0.33	0.34	– − +	− 0.01	0.71	– – –
cg16110645	94,850,433	5UTR	− 0.64	0.34	0.39	0.67	−++	0.55	0.59	+ − +	0.08	0.85	+ − +	−0.02	0.75	– − +
cg10832639	94,850,965	5UTR	−0.25	0.72	1.24	0.16	+++	0.01	0.99	−++	0.18	0.61	−++	0.00	0.95	++−
cg02126235	94,855,034	5UTR	−0.30	0.58	0.76	0.45	+++	0.67	0.55	++−	− 0.26	0.55	– – –	0.02	0.69	++−
cg22828990	94,855,099	5UTR	−0.09	0.82	0.19	0.79	– − +	0.30	0.68	– − +	0.69	0.03	+ − +	**−0.09**	**0.04**	– – –
cg09968361	94,855,344	5UTR	−0.33	0.44	**1.43**	**0.04**	+++	0.65	0.36	−++	**0.72**	**0.02**	+++	**−0.10**	**0.01**	–
cg13826459	94,855,505	TSS1500	−0.08	0.76	0.16	0.76	−++	**0.98**	**0.05**	+++	−0.05	0.83	− + −	0.04	0.15	+++
cg25968219	94,855,590	TSS1500	**−1.81**	**0.05**	**3.29**	**0.003**	+++	−1.29	0.37	– − +	0.37	0.59	++−	− 0.08	0.15	– − +
cg01606800	94,855,990	TSS1500	**−1.08**	**0.001**	0.47	0.47	−++	−0.13	0.85	– − +	− 0.11	0.73	– − +	− 0.01	0.75	– − +
cg02181506	94,856,984	1stExon	−0.78	0.08	0.11	0.89	– − +	0.65	0.40	+ − +	0.28	0.47	+ − +	0.02	0.71	+ − –
cg10070185	94,857,151	TSS200	−0.05	0.91	0.37	0.59	– − +	1.08	0.16	+++	0.24	0.39	−++	0.04	0.29	+ − +
cg24621042	94,857,275	TSS1500	**−0.68**	**0.04**	0.87	0.14	+++	1.16	0.06	+++	−0.15	0.60	− + −	− 0.01	0.87	+ − +
cg00802237	94,858,066	TSS1500	−0.07	0.81	−0.01	0.99	+ − –	− 0.48	0.40	– − +	0.12	0.67	+ − +	−0.03	0.35	– − +
cg04179148	94,858,339	TSS1500	−0.70	0.06	**1.80**	**0.01**	+++	0.29	0.67	+++	0.36	0.25	+++	−0.03	0.47	– – –

Cross-sectional and repeat cross-sectional models adjusted for: study center, age, age2, education, height, height2, sex, sex*age, (sex*age) 2, sex*height, (sex*height)2, Bcell, CD4T, CD8T, Eos, Mono, NK. Repeat cross-sectional in addition ran with a random intercept on the subject. In predictive models, annual change in lung function was computed as the difference between T2 and T1 divided by time of follow-up: (T2-T1)/follow-up models adjusted for covariates from T1

Sample size for circulating AAT for SAPALDIA *n* = 561. Cross-sectional and prediction models (change) *n* = 1076, and *n* = 1122 for repeat cross-sectional models

Meta-Analysis of Lung Function Association of Methylation of CpGs in the SERPINA1* Gene with FEV1 Level and Decline in Adult Ever-Smokers from SAPALDIA, ECRHS and NFBC and with Circulating AAT Concentrations in SAPALDIA

From SERPINA gene cluster located on chromosome 14 between positions: 94′641’781 and 95′235’125 (human genome build 37/hg19)

^a^Nominal *P*-values, Bonferroni corrected significance level for 119 tests is *P*-value 4.6 × 10^− 4^

^b^Direction of effect for SAPALDIA, ECRHS and NFBC. A positive sign indicates that an increase in methylation is associated with higher level of lung function (cross-sectional models) and with an attenuation of lung function decline, respectively (change model)

Statistically significant results are highlighted in bold

**Table 3:** Results from meta-analysis, FEV_1/_FVC and AAT, adult cohorts

CpG	Position	Gene Group	SAPALDIA		Meta- Analysis of Lung Function Association in SAPALDIA, NFBC and ECRHS											
			Circulating AAT		Cross-Sectional T1			Cross-Sectional T2			Repeat Cross-Sectional			Change		
			Coef.	*P*.value^a^	Coef.	*P*.value^a^	Direction^b^	Coef.	*P*.value^a^	Direction^b^	Coef.	*P*.value^a^	Direction^b^	Coef.	*P*.value^a^	Direction^b^
cg10761141	94,844,776	3UTR	−0.30	0.25	0.10	0.14	−++	−0.02	0.79	+ − +	−0.01	0.79	– − +	0.00	0.82	+ − –
cg25042671	94,849,061	Body	**−0.59**	**0.04**	−0.09	0.29	− + −	− 0.12	0.22	– – –	**−0.12**	**0.03**	+ − –	0.00	0.62	+ − +
cg20267408	94,849,703	5UTR	−0.44	0.21	−0.05	0.57	– – –	0.05	0.59	−++	0.01	0.88	+ − +	0.00	0.78	−++
cg26938334	94,849,747	5UTR	1.16	0.42	0.10	0.24	− + −	−0.10	0.47	−++	0.01	0.84	++−	−0.01	0.33	– − +
cg10832639	94,850,965	5UTR	−0.25	0.72	0.16	0.14	+ − +	0.02	0.86	− + −	0.08	0.18	+++	0.01	0.32	+++
cg02126235	94,855,034	5UTR	−0.30	0.58	0.26	0.06	+++	0.06	0.71	− + −	0.06	0.41	++−	0.00	0.90	– − +
cg09968361	94,855,344	5UTR	−0.33	0.44	0.11	0.23	+++	0.00	0.99	++−	0.05	0.25	++−	−0.01	0.37	– − +
cg13826459	94,855,505	TSS1500	−0.08	0.76	0.07	0.34	+ − +	**0.15**	**0.05**	+ − +	0.04	0.32	+++	0.01	0.24	−++
cg25968219	94,855,590	TSS1500	**−1.81**	**0.05**	**0.31**	**0.02**	+++	−0.16	0.43	– − +	− 0.01	0.90	−++	0.00	0.59	+ − +
cg01606800	94,855,990	TSS1500	**−1.08**	**0.001**	**0.17**	**0.05**	+ − +	0.01	0.96	– − +	0.00	0.97	– − +	0.00	0.73	++−
cg02181506	94,856,984	1stExon	−0.78	0.08	−0.01	0.89	+ − –	0.05	0.64	+ − –	−0.03	0.58	+ − –	0.00	0.80	++−
cg10070185	94,857,151	TSS200	−0.05	0.91	0.07	0.45	+++	0.19	0.09	−++	−0.01	0.81	– − +	0.00	0.63	– − +
cg24621042	94,857,275	TSS1500	**−0.68**	**0.04**	**0.18**	**0.02**	+++	0.16	0.08	+++	0.07	0.09	+++	−0.01	0.09	+ − –
cg00802237	94,858,066	TSS1500	−0.07	0.81	0.03	0.71	+ − +	−0.14	0.09	– – –	0.00	0.96	−++	0.00	0.50	+ − –
cg04179148	94,858,339	TSS1500	−0.70	0.06	**0.20**	**0.03**	+++	0.04	0.73	++−	**0.12**	**0.02**	+++	0.00	0.57	– – –

Cross-Sectional and repeat cross-sectional models adjusted for: study center, age, age2, education, height, height2, sex, sex*age, (sex*age) 2, sex*height, (sex*height)2, Bcell, CD4T, CD8T, Eos, Mono, NK. Repeat cross-sectional in addition ran with a random intercept on the subject. In predictive models, annual change in lung function was computed as the difference between T2 and T1 divided by time of follow-up: (T2-T1)/follow-up, models adjusted for covariates from T1

Sample size for circulating AAT for SAPALDIA n = 561. Cross-sectional and prediction models (change) *n* = 1076, and *n* = 1122 for repeat cross-sectional models

Results from Meta-Analysis of Lung Function Association of Methylation of CpGs in the SERPINA1* Gene with FEV1/FVC Level and Decline in Adult Ever-Smokers from SAPALDIA, ECRHS and NFBC and with Circulating AAT Concentrations in SAPALDIA

From SERPINA gene cluster located on chromosome 14 between positions: 94′’641’781 and 95′’235’125 (human genome build 37/hg19)

^a^Nominal *P*-values, Bonferroni corrected significance level for 119 tests is P-value 4.6 × x10^-4^

^b^Direction of effect for SAPALDIA, ECRHS and NFBC. A positive sign indicates that an increase in methylation is associated with higher level of lung function (cross-sectional models) and with an attenuation of lung function decline, respectively (change model)

Statistically significant results are highlighted in bold

#### SERPINA gene cluster

Results from the meta-analysis on the association of methylation at the 119 CpGs with cross-sectional lung function and lung function decline for FEV_1_, FVC and FEV_1_/FVC are presented in Additional file 1: Tables ES2-S4. A single CpG at cg08257009, located 32 kb downstream of *SERPINA1*, withstood Bonferroni-correction for multiple testing. Methylation at this site was positively associated with FEV_1_/FVC in the repeat cross-sectional analysis (*β:*0.11; *P*-value = 2.6 × 10^− 4^) (Additional file 1: Table ES3). The associations of this signal for the two cross-sectional time points were comparable (*β*:0.10; *P*-value = 0.01 at T1; *β*:0.14; *P*-value = 2.2 × 10^− 3^ at T2). Consistent with the observation that higher methylation at this site was associated with higher level of FEV_1_/FVC cross-sectionally, it also predicted attenuated decline of FEV_1_/FVC (*β*:0.0089; *P*-value = 9.1 × 10^− 3^).

### Association of methylation at 119 CpGs with circulating alpha-1 antitrypsin in adult ever smokers in SAPALDIA

None of the associations between methylation and circulating AAT in SAPALDIA participants reached multiple-testing-corrected statistical significance, but nominal statistical significance was observed at four CpG sites in the *SERPINA1* gene (Tables 2 and 3) and at two additional sites outside *SERPINA1* (Additional file 1: Tables ES2-S4), one of which was cg08257009, the only lung function associated signal withstanding multiple testing. In all instances, higher methylation was associated with lower AAT concentrations. These inverse associations with circulating AAT did not translate into statistically significant and inverse associations with any of the lung function parameters measured.

### Association of methylation at 119 CpGs with lung function in tobacco-smoke exposed children and adolescents

Characteristics of ALSPAC children and adolescents from the first (T1) and second (T2) time points are presented in Table 4. Height and weight increased during follow-up from a mean of 133 to 170 cm and from 31 to 63 kg, respectively. Twenty-two and 29% of children and adolescents had asthma and at the second time point, the majority (86%) of adolescents reported intake of asthma medication in the last 12 months. While FEV_1_ and FVC increased during follow-up, on average, FEV_1_/FVC remained stable.

**Table 4:** Characteristics of tobacco-smoke exposed children and adolescents^a^, ALSPAC

	Children Cross-Sectional T1 (2000)	Adolescents Cross-Sectional T2 (2007)
N	259	259
Females (%)	53.7	53.7
Age (years), mean (SD^b^)	8.6 (0.2)	15.4 (0.3)
Height (cm), mean (SD)	133.3 (6.0)	170.2 (8.0)
Weight (kg), mean (SD)	31.3 (6.3)	62.8 (11.0)
BMI	17.4 (2.5)	21.7 (3.3)
Maternal Education (%)		
Ordinary Level or Lower	56.4	56.4
Advanced Level	27.8	27.8
University	15.8	15.8
Asthmatics (%)	21.6	28.9
Smoking Exposure (%)		
In-utero smoking (smoking mother)	25.1	25.1
Second-hand exposure at home	56.0	56.0
Self-exposure (smoked > 2 times in a life-time)	52.1	52.1
COPD [FEV_1_/FVC < LLN^c^] (%)	5.0	4.6
Asthma medications in the last 12 months (%)	14.0	14.0
FVC(L), mean (SD)	2.0 (0.3)	3.9 (0.9)
FEV_1_(L), mean (SD)	1.7 (0.3)	3.4 (0.7)
FEV_1_/FVC(L), mean (SD)	0.9 (0.06)	0.9 (0.07)

^a^Children and adolescents exposed to tobacco-smoke defined as: mother smoked during pregnancy and/or lived with a smoker and/or reported smoking ≥ twice in their lifetime

^b^SD, standard deviation

^c^LLN, lower limit of normal estimated using GLI2012 reference equations

#### SERPINA1

Focusing on the methylation sites in *SERPINA1* (Additional file 1: Tables ES5-S7), again none of the signals reached Bonferroni-corrected significance in association with any lung function parameter or model. There were several nominally statistically significant associations, particularly for lung function growth (FEV_1_: cg26938334; FVC: cg26938334, cg13826459, cg 24,621,042; FEV_1_/FVC: cg10070185). Methylation at cg26938334 was inversely associated with growth in FEV_1_ and FVC, and methylation at cg24621042 and cg10070185 was inversely associated with growth in FVC and in FEV_1_/FVC, respectively. The associations of cg24621042 with FEV1/FVC change were inconsistent with those observed for FEV1/FVC decline in adults.

#### SERPINA gene cluster

Results from meta-analysis on the association of methylation at all 119 CpGs with cross-sectional lung function and lung function growth of FEV_1_, FVC and FEV_1_/FVC are presented in Additional file 1: Tables ES5-S7. No methylation signals remained statistically significant after Bonferroni correction. Methylation at cg08257009, 32 kb downstream of *SERPINA1* gene, which showed statistically significant positive association with FEV_1_/FVC cross-sectionally in the adult cohort, was not associated with FEV_1_ or FEV_1_/FVC in children. However, there was evidence for an inverse, rather than a positive association with FVC at T1 (*β*:-0.91, *P*-value = 4.4 × 10^− 3^, Additional file 1: Table ES6). Overall, none of the *SERPINA*1 CpGs showed consistent associations across cross-sectional and longitudinal models.

### Replication of previously reported COPD signals

Relative hypomethylation of cg02181506 and cg24621042 were previously associated with COPD [9]. Table 5 summarizes the association of both signals with circulating AAT in SAPALDIA and with lung function and its change in adults as well as children. Contrary to expectation, relative hypermethylation, not hypomethylation at these two sites was associated with lower circulating AAT. For cg02181506, irrespective of age, no association of methylation with either FEV_1_/FVC or FEV_1_ was observed. For cg24621042, in adults, hypermethylation was positively associated with FEV_1_/FVC at T1 only, but not in the repeat cross-sectional analysis, and the inverse association of methylation at cg24621042 and FVC change was only observed in children. No lung function associations reached statistical significance at the nominal *P*-value< 0.05.

**Table 5:** Association of previously reported COPD associated cg02181506 and cg2462102

Circulating AAT^a^	cg02181506		cg24621042	
	Coef.	*P*_value_	Coef.	*P*_value_
	− 0.78	0.08	− 0.68	0.04
Adults (SAPALDIA, NFBC66, ECRHS)				
FEV_1_, repeat cross-sectional	0.28	0.47	−0.15	0.60
FEV_1_, change	0.02	0.71	−0.01	0.87
Children and Adolescents (ALSPAC)				
FEV_1_, repeat cross-sectional	1.42	0.35	0.31	0.29
FEV_1_, change	−0.54	0.26	− 0.17	0.06
Adults (SAPALDIA, NFBC66, ECRHS)				
FEV_1_/FVC, repeat cross-sectional	−0.03	0.58	0.07	0.09
FEV_1_/FVC, change	0.00	0.80	−0.01	0.09
Children and Adolescents (ALSPAC)				
FEV_1_/FVC, repeat cross-sectional	0.01	0.98	0.08	0.07
FEV_1_/FVC, change	−0.12	0.06	0.00	0.71

Direction of effect for SAPALDIA, ECRHS and NFBC: a positive sign indicates that an increase in methylation is associated with higher level of lung function (cross-sectional models) and with an attenuation of lung function decline in the prediction models (change in lung function). In ALSPAC children, a positive sign indicates that an increase in methylation is associated with higher level of lung function (cross-sectional models) and with lung function growth in the prediction models (change in lung function)

Association of Previously Reported COPD Associated cg02181506 and cg2462102 with Circulating AAT and Lung Function in Adults, Children and Adolescents

^a^ SAPALDIA cohort, first time point

### Sensitivity analyses in the SAPALDIA cohort

In the absence of consistent CpG and lung function associations, we restricted sensitivity analyses to the FEV_1_/FVC association between the CpG withstanding multiple testing (cg08257009) and the two CpGs (cg02181506 and cg24621042) previously associated with COPD [9] (Table 6, Additional file 1: Tables ES8-S9). Sensitivity analyses did not reveal a more consistent pattern of associations between methylation at these three sites and lung function.

**Table 6:** Sensitivity Analyses of Association between FEV_1_/FVC and Methylation at cg02181506, cg2462102, and cg08257009, SAPALDIA Cohort

	cg02181506		cg24621042		cg08257009	
	Coef.	*P*_value_	Coef.	*P*_value_	Coef.	*P*_value_
FEV_1_/FVC repeat cross-sectional						
Main model^a^	−0.01	0.93	0.05	0.54	0.07	0.12
Adjustment for PiS and PiZ genotype	−0.01	0.94	0.05	0.54	0.07	0.12
Adjustment for neutrophils at T1 and T2	−0.00	0.97	0.05	0.52	0.07	0.11
Men	0.03	0.86	0.07	0.47	0.10	0.11
Women	−0.07	0.65	0.06	0.62	0.05	0.49
Not obese (BMI < 30 kg/m^2^)	−0.06	0.58	0.04	0.60	**0.12**	**0.01**
Obese (BMI ≥ 30 kg/m^2^)	0.19	0.25	0.06	0.68	−0.12	0.14
No self-report of ever asthma at T1 and T2	−0.03	0.78	0.07	0.35	0.08	0.09
Asthma ever self-report at T1 or T2	0.14	0.44	−0.05	0.72	−0.01	0.88
FEV_1_/FVC change^b^						
Main model^c^	−0.01	0.60	−0.01	0.49	**0.02**	**0.005**
Adjustment for circulating hs-crp and AAT^d^	−0.008	0.56	−0.007	0.01	**0.02**	**0.006**
Adjustment for PiS and PiZ genotype	−0.01	0.57	−0.01	0.48	**0.02**	**0.005**
Adjustment for neutrophils at T1 and T2	−0.01	0.44	−0.01	0.36	**0.02**	**0.01**
Men	0.01	0.77	−0.00	0.74	**0.02**	**0.01**
Women	−0.03	0.14	−0.01	0.56	0.02	0.08
Not obese (BMI < 30 kg/m^2^) at T1	−0.00	0.84	−0.00	0.87	**0.02**	**0.01**
Obese (BMI ≥ 30 kg/m^2^) at T1	−0.04	0.13	−0.04	0.07	0.02	0.17
No self-report of ever asthma at T1 and T2	−0.01	0.57	−0.01	0.40	**0.01**	**0.04**
Asthma ever self-report at T1 or T2	−0.03	0.28	0.01	0.65	**0.03**	**0.04**

^a^Adjusted for: study center, age, age^2^, education, height, height^2^, sex, sex*age, (sex*age)^2^, sex*height, (sex*height)^2^, Bcell, CD4T, CD8T, Eos, Mono, NK and ran with a random intercept on the subject

^b^Direction of effect. A positive sign indicates that an increase in methylation is associated with higher level of lung function (cross-sectional models) and with an attenuation of lung function decline in the prediction models (change in lung function)

^c^Adjusted for covariates at T1: study center, age, age^2^, education, height, height^2^, sex, sex*age, (sex*age)^2^, sex*height, (sex*height)^2^, Bcell, CD4T, CD8T, Eos, Mono, NK. Change in lung function was computed as the difference between T2 and T1 divided by time of follow-up: (T2-T1)/follow-up

^d^Circulating hs-crp and AAT available from T1 only

Statistically significant results are highlighted in bold

## Discussion

This is the first study to investigate the association of DNAm in the *SERPINA* gene cluster with lung function and its longitudinal change in ever-smoking adults from three European population-based adult cohort studies and in tobacco-smoke exposed children and adolescents from England. We observed methylation at cg08257009 not annotated to a gene in the *SERPINA* gene cluster, located 32 kb downstream of the *SERPINA1* gene to be significantly associated with FEV_1_/FVC at the Bonferroni-corrected level. No methylation signals in the *SERPINA1* gene showed associations with lung function level or change over time after correcting for multiple testing.

Few studies have reported associations between DNAm and lung function or COPD. Our results obtained in general population samples contradict the previous finding of hypomethylation at two CpG sites in *SERPINA1* and COPD risk in two family-based studies. The first of these studies was restricted to smokers and the second consisted of participants with and without a history of smoking [9]. The functional relevance of the two CpG sites remains unclear, given that the associations of hypomethylation with COPD risk and circulating AAT are inconsistent in direction. Consistent with the inverse association observed with AAT in the blood, hypomethylation of the AAT gene has been associated with increased gene expression in rat models [26].

Our results are consistent with the results from a study comparing gene methylation in lung tissue of former smoker COPD patients and controls with normal lung function [27, 28]. Methylation in *SERPINA1* was not associated with COPD risk. Similarly no cross-sectional association with post-bronchodilation lung function was observed for *SERPINA1* gene methylation measured in blood samples from a small rural Korean COPD case-control study [29]. Furthermore, in a study of middle-aged monozygotic twins, DNAm in *SERPINA1* was not associated with intra-pair differences in lung function decline [30].

Smoking exerts strong adverse effects on lung function and increases COPD risk. In a small proportion of smokers, it interacts with genetically determined rare AAT deficiency to cause COPD. The adverse respiratory effects on the lung tissue may therefore be mediated by altering DNAm in *SERPINA1*. Yet, the largest epigenome-wide association study for smoking did not identify a relevant association between epigenetic signatures in *SERPINA1* and smoking [31]. Interestingly, the cg08257009 which reached Bonferroni corrected significance in the repeat cross-sectional analysis was previously reported as a smoking-related CpG in buccal cells in epithelial cancer [32]. In addition, cg08257009 and cg02181506 were reported to be smoking related signals in blood-derived DNA samples from participants in 16 cohorts, with cg02181506 showing weaker associations [31].

The use of blood instead of lung tissue to assess lung disease related methylation may be considered a limitation of the study. However as previously discussed, COPD is a systemic disease related to low grade inflammation, which supports studying the peripheral blood methylome [9]. Teschendorff and others demonstrated the correlation between smoking related DNAm in blood and lung tissue as well as between normal and malignant lung tissue. Their results also pointed to the prognostic value of these signals in lung cancer patients [32–34].

Additional limitations of the study include the lack of cross–omics data to investigate the biological network related to AAT more comprehensively. This is important, given the fact that the most recent lung function GWAS suggests an important role of elastic-fibre pathways [7]. Furthermore, misclassification of smoking exposure could have biased the observed associations, most likely towards the null.

The advantages of the current study are several fold. First, the studies providing data for this investigation are well established respiratory population-based cohorts. They are known for high quality testing of lung function and large sample sizes. They share similarities in study protocols. Methylome analyses followed stringent quality control. Second, the study is based on longitudinal lung function data and prospectively and repeatedly measured DNAm. Repeated assessment of lung function and predictors may have helped improve the statistical power of the cross-sectional analysis. Prospective assessment of DNAm with change in lung function decreases the problem of reverse causation. Third, the integration of lung function from both adults and children offered the opportunity to study *SERPINA1* methylation and lung function over the life course and investigate whether relevant time windows for genome-environment interactions may exist.

## Conclusion

In conclusion, this first comprehensive study on DNAm in the *SERPINA* gene cluster provides weak evidence of an association with lung functions and its change across the life course. Larger studies based on post-bronchodilation lung function need to be followed by investigating associations of *SERPINA1* and elastin-related pathways and networks through cross–omics approaches.

## Additional file

Additional file 1: **Table ES1.** Meta-Analysis of the association of methylation at CpGs in the SERPINA* Gene Cluster with FVC level and decline in adult ever smokers from SAPALDIA, ECRHS and NFBC (*n* = 1076) and with circulating AAT concentrations in SAPALDIA (*n* = 561). **Table ES2.** Meta-Analysis of the association of methylation at 119 CpGs in the SERPINA* cluster with FEV_1_ level and decline in adult ever smokers from SAPALDIA, ECRHS and NFBC (n = 1076) and with circulating AAT concentrations in SAPALDIA (n = 561). **Table ES3.** Meta-analysis of the association of methylation at 119 CpGs in the SERPINA* gene cluster with FVC level and decline in adult ever smokers from SAPALDIA, ECRHS and NFBC (n = 1076) and with circulating AAT concentrations in SAPALDIA (n = 561). **Table ES4.** Meta-analysis of the association of methylation at 119 CpGs in the SERPINA* gene cluster with FEV_1_/FVC level and decline in adult ever smokers from SAPALDIA, ECRHS and NFBC (n = 1076) and with circulating AAT concentrations in SAPALDIA (n = 561). **Table ES5.** Association of methylation at 119 CpGs in the SERPINA* gene cluster with FEV_1_ level and decline in ALSPAC children exposed to tobacco smoke (*n* = 259). **Table ES6.** Association of methylation at 119 CpGs in the SERPINA* gene cluster with FVC level and decline in in ALSPAC children exposed to tobacco smoke (n = 259). **Table ES7.** Association of methylation at 119 CpGs in the SERPINA* gene cluster with FEV_1_/FVC level and decline in ALSPAC children exposed to tobacco smoke (*n* = 259). **Table ES8.** Association of methylation at 119 CpGs in the SERPINA* gene cluster with FEV_1_ level and decline in adult smokers from SAPALDIA, basic adjustment and adjustment for PIS and PIZ genotypes. **Table ES9.** Association of methylation at 119 CpGs in the SERPINA* gene cluster with FEV_1_/FVC level and decline in adult smokers from SAPALDIA, basic adjustment and adjustment for PIS and PIZ genotypes. (DOCX 349 kb)

## References

[1] Salvi SS, Barnes PJ (2009). Chronic obstructive pulmonary disease in non-smokers. Lancet.

[2] Hazari YM, Bashir A, Habib M, Bashir S, Habib H, Qasim MA, Shah NN, Haq E, Teckman J, Fazili KM (2017). Alpha-1-antitrypsin deficiency: genetic variations, clinical manifestations and therapeutic interventions. Mutat Res.

[3] Sandhaus RA, Stoller JK (2013). Introduction to the 50th anniversary of the description of alpha-1 antitrypsin deficiency. COPD.

[4] Thun GA, Imboden M, Ferrarotti I, Kumar A, Obeidat M, Zorzetto M, Haun M, Curjuric I, Couto Alves A, Jackson VE (2013). Causal and synthetic associations of variants in the SERPINA gene cluster with alpha1-antitrypsin serum levels. PLoS Genet.

[5] Busch R, Hobbs BD, Zhou J, Castaldi PJ, McGeachie MJ, Hardin ME, Hawrylkiewicz I, Sliwinski P, Yim JJ, Kim WJ (2017). Genetic association and risk scores in a chronic obstructive pulmonary disease Meta-analysis of 16,707 subjects. Am J Respir Cell Mol Biol.

[6] Lutz SM, Cho MH, Young K, Hersh CP, Castaldi PJ, McDonald ML, Regan E, Mattheisen M, DeMeo DL, Parker M (2015). A genome-wide association study identifies risk loci for spirometric measures among smokers of European and African ancestry. BMC Genet.

[7] Wain LV, Shrine N, Artigas MS, Erzurumluoglu AM, Noyvert B, Bossini-Castillo L, Obeidat M, Henry AP, Portelli MA, Hall RJ (2017). Genome-wide association analyses for lung function and chronic obstructive pulmonary disease identify new loci and potential druggable targets. Nat Genet.

[8] Corley M, Solem A, Phillips G, Lackey L, Ziehr B, Vincent HA, Mustoe AM, Ramos SBV, Weeks KM, Moorman NJ, Laederach A (2017). An RNA structure-mediated, posttranscriptional model of human alpha-1-antitrypsin expression. Proc Natl Acad Sci U S A.

[9] Qiu W, Baccarelli A, Carey VJ, Boutaoui N, Bacherman H, Klanderman B, Rennard S, Agusti A, Anderson W, Lomas DA, DeMeo DL (2012). Variable DNA methylation is associated with chronic obstructive pulmonary disease and lung function. Am J Respir Crit Care Med.

[10] Martin BW, Ackermann-Liebrich U, Leuenberger P, Kunzli N, Stutz EZ, Keller R, Zellweger JP, Wuthrich B, Monn C, Blaser K (1997). SAPALDIA: methods and participation in the cross-sectional part of the Swiss study on air pollution and lung diseases in adults. Soz Praventivmed.

[11] Ackermann-Liebrich U, Kuna-Dibbert B, Probst-Hensch NM, Schindler C, Felber Dietrich D, Stutz EZ, Bayer-Oglesby L, Baum F, Brandli O, Brutsche M (2005). Follow-up of the Swiss Cohort Study on Air Pollution and Lung Diseases in Adults (SAPALDIA 2) 1991-2003: methods and characterization of participants. Soz Praventivmed.

[12] Janson C, Anto J, Burney P, Chinn S, de Marco R, Heinrich J, Jarvis D, Kuenzli N, Leynaert B, Luczynska C (2001). The European Community respiratory health survey: what are the main results so far? European Community respiratory health survey II. Eur Respir J.

[13] University of Oulu. Northern Finland Cohorts. 31-year follow-up study. 2018. http://www.oulu.fi/nfbc/node/18097. Accessed 18 Mar 2018.

[14] Boyd A, Golding J, Macleod J, Lawlor DA, Fraser A, Henderson J, Molloy L, Ness A, Ring S, Davey Smith G (2013). Cohort profile: the ‘children of the 90s’--the index offspring of the Avon longitudinal study of parents and children. Int J Epidemiol.

[15] Miller MR, Hankinson J, Brusasco V, Burgos F, Casaburi R, Coates A, Crapo R, Enright P, van der Grinten CP, Gustafsson P (2005). Standardisation of spirometry. Eur Respir J.

[16] Bridevaux PO, Dupuis-Lozeron E, Schindler C, Keidel D, Gerbase MW, Probst-Hensch NM, Bettschart R, Burdet L, Pons M, Rothe T (2015). Spirometer replacement and serial lung function measurements in population studies: results from the SAPALDIA study. Am J Epidemiol.

[17] Kotecha SJ, Watkins WJ, Lowe J, Henderson AJ, Kotecha S (2016). Effect of early-term birth on respiratory symptoms and lung function in childhood and adolescence. Pediatr Pulmonol.

[18] Aryee MJ, Jaffe AE, Corrada-Bravo H, Ladd-Acosta C, Feinberg AP, Hansen KD, Irizarry RA (2014). Minfi: a flexible and comprehensive bioconductor package for the analysis of Infinium DNA methylation microarrays. Bioinformatics.

[19] Teschendorff AE, Marabita F, Lechner M, Bartlett T, Tegner J, Gomez-Cabrero D, Beck S (2013). A beta-mixture quantile normalization method for correcting probe design bias in Illumina Infinium 450 k DNA methylation data. Bioinformatics.

[20] Assenov Y, Muller F, Lutsik P, Walter J, Lengauer T, Bock C (2014). Comprehensive analysis of DNA methylation data with RnBeads. Nat Methods.

[21] Lehne B, Drong AW, Loh M, Zhang W, Scott WR, Tan ST, Afzal U, Scott J, Jarvelin MR, Elliott P (2015). A coherent approach for analysis of the Illumina HumanMethylation450 BeadChip improves data quality and performance in epigenome-wide association studies. Genome Biol.

[22] Touleimat N, Tost J (2012). Complete pipeline for Infinium((R)) human methylation 450K BeadChip data processing using subset quantile normalization for accurate DNA methylation estimation. Epigenomics.

[23] Houseman EA, Accomando WP, Koestler DC, Christensen BC, Marsit CJ, Nelson HH, Wiencke JK, Kelsey KT (2012). DNA methylation arrays as surrogate measures of cell mixture distribution. BMC Bioinformatics.

[24] Senn O, Russi EW, Schindler C, Imboden M, von Eckardstein A, Brandli O, Zemp E, Ackermann-Liebrich U, Berger W, Rochat T (2008). Circulating alpha1-antitrypsin in the general population: determinants and association with lung function. Respir Res.

[25] Willer CJ, Li Y, Abecasis GR (2010). METAL: fast and efficient meta-analysis of genomewide association scans. Bioinformatics.

[26] Barton DE, Francke U (1987). Activation of human alpha 1-antitrypsin genes in rat hepatoma x human fibroblast hybrid cell lines is correlated with demethylation. Somat Cell Mol Genet.

[27] Morrow JD, Glass K, Cho MH, Hersh CP, Pinto-Plata V, Celli B, Marchetti N, Criner G, Bueno R, Washko G, et al. Human lung DNA methylation quantitative trait loci Colocalize with COPD genome-wide association loci. Am J Respir Crit Care Med. 2018;197:1275–84. 10.1164/rccm.201707-1434OC PMC5955059 29313708

[28] Morrow JD, Cho MH, Hersh CP, Pinto-Plata V, Celli B, Marchetti N, Criner G, Bueno R, Washko G, Glass K, et al. DNA methylation profiling in human lung tissue identifies genes associated with COPD. Epigenetics. 2016;11:730–9. 10.1080/15592294.2016.1226451 PMC5094634 27564456

[29] Lee MK, Hong Y, Kim SY, Kim WJ, London SJ (2017). Epigenome-wide association study of chronic obstructive pulmonary disease and lung function in Koreans. Epigenomics.

[30] Bolund ACS, Starnawska A, Miller MR, Schlunssen V, Backer V, Borglum AD, Christensen K, Tan Q, Christiansen L, Sigsgaard T (2017). Lung function discordance in monozygotic twins and associated differences in blood DNA methylation. Clin Epigenetics.

[31] Joehanes R, Just AC, Marioni RE, Pilling LC, Reynolds LM, Mandaviya PR, Guan W, Xu T, Elks CE, Aslibekyan S (2016). Epigenetic signatures of cigarette smoking. Circ Cardiovasc Genet.

[32] Teschendorff AE, Yang Z, Wong A, Pipinikas CP, Jiao Y, Jones A, Anjum S, Hardy R, Salvesen HB, Thirlwell C (2015). Correlation of smoking-associated DNA methylation changes in Buccal cells with DNA methylation changes in epithelial Cancer. JAMA Oncol.

[33] Stueve TR, Li WQ, Shi J, Marconett CN, Zhang T, Yang C, Mullen D, Yan C, Wheeler W, Hua X (2017). Epigenome-wide analysis of DNA methylation in lung tissue shows concordance with blood studies and identifies tobacco smoke-inducible enhancers. Hum Mol Genet.

[34] Russo AL, Thiagalingam A, Pan H, Califano J, Cheng KH, Ponte JF, Chinnappan D, Nemani P, Sidransky D, Thiagalingam S (2005). Differential DNA hypermethylation of critical genes mediates the stage-specific tobacco smoke-induced neoplastic progression of lung cancer. Clin Cancer Res.

