# Supplementary material for: SERPINA1 methylation and lung function in tobacco-smoke exposed European children and adults: a meta-analysis of ALEC population-based cohorts
Source: Respir Res. 2018 Aug 22;19:156. doi: 10.1186/s12931-018-0850-8 (PMC6103990; doi:10.1186/s12931-018-0850-8)
Supplement: Supplementary file 1 — Table ES1. Meta-Analysis of the association of methylation at CpGs in the SERPINA* Gene Cluster with FVC level and decline in adult ever smokers from SAPALDIA, ECRHS and NFBC (n = 1076) and with circulating AAT concentrations in SAPALDIA (n = 561). Table ES2. Meta-Analysis of the association of methylation at 119 CpGs in the SERPINA* cluster with FEV1 level and decline in adult ever smokers from SAPALDIA, ECRHS and NFBC (n = 1076) and with circulating AAT concentrations in SAPALDIA (n = 561). Table ES3. Meta-analysis of the association of methylation at 119 CpGs in the SERPINA* gene cluster with FVC level and decline in adult ever smokers from SAPALDIA, ECRHS and NFBC (n = 1076) and with circulating AAT concentrations in SAPALDIA (n = 561). Table ES4. Meta-analysis of the association of methylation at 119 CpGs in the SERPINA* gene cluster with FEV1/FVC level and decline in adult ever smokers from SAPALDIA, ECRHS and NFBC (n = 1076) and with circulating AAT concentrations in SAPALDIA (n = 561). Table ES5. Association of methylation at 119 CpGs in the SERPINA* gene cluster with FEV1 level and decline in ALSPAC children exposed to tobacco smoke (n = 259). Table ES6. Association of methylation at 119 CpGs in the SERPINA* gene cluster with FVC level and decline in in ALSPAC children exposed to tobacco smoke (n = 259). Table ES7. Association of methylation at 119 CpGs in the SERPINA* gene cluster with FEV1/FVC level and decline in ALSPAC children exposed to tobacco smoke (n = 259). Table ES8. Association of methylation at 119 CpGs in the SERPINA* gene cluster with FEV1 level and decline in adult smokers from SAPALDIA, basic adjustment and adjustment for PIS and PIZ genotypes. Table ES9. Association of methylation at 119 CpGs in the SERPINA* gene cluster with FEV1/FVC level and decline in adult smokers from SAPALDIA, basic adjustment and adjustment for PIS and PIZ genotypes. (DOCX 349 kb) [file 12931_2018_850_MOESM1_ESM.docx]

**Submission to Respiratory Research – ONLINE DATA SUPPLEMENT**

May 13th 2018

***SERPINA1* Methylation and Lung Function in Tobacco-Smoke Exposed European Children and Adults: a Meta-Analysis of ALEC population-based cohorts.**

Anna Beckmeyer-Borowko*, Medea Imboden*, Faisal I. Rezwan*, Matthias Wielscher*, Andre F.S. Amaral*, Ayoung Jeong, Emmanuel Schaffner, Juha Auvinen, Sylvain Sebert, Ville Karhunen, Robert Bettschart, Alexander Turk, Marco Pons, Daiana Stolz, Florian Kronenberg, Ryan Arathimos, Gemma C Sharp, Caroline Relton, Alexander J. Henderson, Marjo-Riitta Jarvelin**, Deborah Jarvis^**^, John W. Holloway**, Nicole M. Probst-Hensch**

*equal first author contribution; **equal senior author contribution

Table ES1: Meta-Analysis of the association of methylation at CpGs in the *SERPINA** Gene Cluster with FVC level and decline in adult ever smokers from SAPALDIA, ECRHS and NFBC (n=1076) and with circulating AAT concentrations in SAPALDIA (n=561)

*From *SERPINA* gene cluster located on chromosome 14 between positions: 94’641’781 and 95’235’125 (human genome build 37/hg19).
† Nominal P-values, Bonferroni corrected significance level for 119 tests is P-value 4.6x10^-4^
‡Direction of effect for SAPALDIA, ECRHS and NFBC. A positive sign indicates that an increase in methylation is associated with higher level of lung function (cross-sectional models) and with an attenuation of lung function decline, respectively (change model).
Cross-Sectional and repeat cross-sectional models were adjusted for: study center, age, age^2^, education, height, height^2^, sex, sex*age, (sex*age)^2^, sex*height, (sex*height)^2^, Bcell, CD4T, CD8T, Eos, Mono, NK. Repeat cross-sectional in addition ran with a random intercept on the subject. In predictive models, annual change in lung function was computed as the difference between T2 and T1 divided by time of follow-up: (T2-T1)/follow-up models adjusted for covariates from T1
Sample size for circulating AAT for SAPALDIA n= 561, cross-sectional and prediction models (change) n=1076, and n=1122 for repeat cross-sectional models
Significant CpGs at the nominal level are bolded

Table ES2: Meta-Analysis of the association of methylation at 119 CpGs in the *SERPINA** cluster with FEV_1_ level and decline in adult ever smokers from SAPALDIA, ECRHS and NFBC (n=1076) and with circulating AAT concentrations in SAPALDIA (n=561)

*From *SERPINA* gene cluster located on chromosome 14 between positions: 94’641’781 and 95’235’125 (human genome build 37/hg19).
† Nominal P-values, Bonferroni corrected significance level for 119 tests is P-value 4.6x10^-4^
‡Direction of effect for SAPALDIA, ECRHS and NFBC. A positive sign indicates that an increase in methylation is associated with higher level of lung function (cross-sectional models) and with an attenuation of lung function decline, respectively (change model).
Cross-Sectional and repeat cross-sectional models were adjusted for: study center, age, age^2^, education, height, height^2^, sex, sex*age, (sex*age)^2^, sex*height, (sex*height)^2^, Bcell, CD4T, CD8T, Eos, Mono, NK. Repeat cross-sectional in addition ran with a random intercept on the subject. In predictive models, annual change in lung function was computed as the difference between T2 and T1 divided by time of follow-up: (T2-T1)/follow-up models adjusted for covariates from T1
Sample size for circulating AAT for SAPALDIA n= 561, cross-sectional and prediction models (change) n= 1076, and n= 1122 for repeat cross-sectional models
*SERPINA1* CpGs are highlighted in bold, italic. Nominally significant CpGs are in addition highlighted in grey
Significant CpGs at the nominal level, outside *SERPINA1* gene, are bolded

Table ES3: Meta-analysis of the association of methylation at 119 CpGs in the *SERPINA** gene cluster with FVC level and decline in adult ever smokers from SAPALDIA, ECRHS and NFBC (n=1076) and with circulating AAT concentrations in SAPALDIA (n=561)

*From *SERPINA* gene cluster located on chromosome 14 between positions: 94’641’781 and 95’235’125 (human genome build 37/hg19).
† Nominal P-values, Bonferroni corrected significance level for 119 tests is P-value 4.6x10^-4^
‡Direction of effect for SAPALDIA, ECRHS and NFBC. A positive sign indicates that an increase in methylation is associated with higher level of lung function (cross-sectional models) and with an attenuation of lung function decline, respectively (change model).
Cross-Sectional and repeat cross-sectional models were adjusted for: study center, age, age^2^, education, height, height^2^, sex, sex*age, (sex*age)^2^, sex*height, (sex*height)^2^, Bcell, CD4T, CD8T, Eos, Mono, NK. Repeat cross-sectional in addition ran with a random intercept on the subject. In predictive models, annual change in lung function was computed as the difference between T2 and T1 divided by time of follow-up: (T2-T1)/follow-up models adjusted for covariates from T1
Sample size for circulating AAT for SAPALDIA n= 561, cross-sectional and prediction models (change) n= 1076, and n= 1122 for repeat cross-sectional models
*SERPINA1* CpGs are highlighted in bold, italic. Nominally significant CpG are in addition highlighted in grey
Significant CpGs at the nominal level, outside *SERPINA1* gene, are bolded

Table ES4: Meta-analysis of the association of methylation at 119 CpGs in the *SERPINA** gene cluster with FEV_1_/FVC level and decline in adult ever smokers from SAPALDIA, ECRHS and NFBC (n=1076) and with circulating AAT concentrations in SAPALDIA (n=561)

*From *SERPINA* gene cluster located on chromosome 14 between positions: 94’641’781 and 95’235’125 (human genome build 37/hg19).
† Nominal P-values, Bonferroni corrected significance level for 119 tests is P-value 4.6x10^-4^
‡Direction of effect for SAPALDIA, ECRHS and NFBC. A positive sign indicates that an increase in methylation is associated with higher level of lung function (cross-sectional models) and with an attenuation of lung function decline, respectively (change model).
Cross-Sectional and repeat cross-sectional models were adjusted for: study center, age, age^2^, education, height, height^2^, sex, sex*age, (sex*age)^2^, sex*height, (sex*height)^2^, Bcell, CD4T, CD8T, Eos, Mono, NK. Repeat cross-sectional in addition ran with a random intercept on the subject. In predictive models, annual change in lung function was computed as the difference between T2 and T1 divided by time of follow-up: (T2-T1)/follow-up models adjusted for covariates from T1
Sample size for circulating AAT for SAPALDIA n= 561, cross-sectional and prediction models (change) n= 1076, and n= 1122 for repeat cross-sectional models
*SERPINA1* CpGs are highlighted in bold, italic. Nominally significant CpGs are in addition highlighted in grey
Significant CpGs at the nominal level, outside *SERPINA1* gene, are bolded.
cg08257009 is bolded and highlighted in dark grey. This is the only CpG in the *SERPINA* cluster that withstood Bonferroni-correction for multiple testing.

Table ES5: Association of methylation at 119 CpGs in the *SERPINA** gene cluster with FEV_1_ level and decline in ALSPAC children exposed to tobacco smoke (n=259)

*From *SERPINA* gene cluster located on chromosome 14 between positions: 94’641’781 and 95’235’125 (human genome build 37/hg19).
† Children and adolescents exposed to tobacco-smoke defined as: mother smoked during pregnancy and/or lived with a smoker and/or reported smoking ≥twice in their lifetime
‡ Nominal P-values, Bonferroni corrected significance level for 119 tests is P-value 4.6x10^-4^
Cross-Sectional and repeat cross-sectional models were adjusted for: study center, age, mother education, height, (height-mean(height))^2^, sex, sex*age, sex*height, and cell composition (CD8T; CD4T; NK; Bcell; Mono; Eos). Repeat cross-sectional in addition ran with a random intercept on the subject. In predictive models, annual change in lung function was computed as the difference between T2 and T1 divided by time of follow-up: (T2-T1)/follow-up models adjusted for covariates from T1
*SERPINA1* CpGs are highlighted in bold, italic. Nominally significant CpGs are in addition highlighted in grey
Significant CpGs at the nominal level, outside *SERPINA1* gene, are bolded.

Table ES6: Association of methylation at 119 CpGs in the *SERPINA** gene cluster with FVC level and decline in in ALSPAC children exposed to tobacco smoke (n=259)

*From *SERPINA* gene cluster located on chromosome 14 between positions: 94’641’781 and 95’235’125 (human genome build 37/hg19).
† Children and adolescents exposed to tobacco-smoke defined as: mother smoked during pregnancy and/or lived with a smoker and/or reported smoking ≥twice in their lifetime
‡ Nominal P-values, Bonferroni corrected significance level for 119 tests is P-value 4.6x10^-4^
Cross-Sectional and repeat cross-sectional models were adjusted for: study center, age, mother education, height, (height-mean(height))^2^, sex, sex*age, sex*height, and cell composition (CD8T; CD4T; NK; Bcell; Mono; Eos). Repeat cross-sectional in addition ran with a random intercept on the subject. In predictive models, annual change in lung function was computed as the difference between T2 and T1 divided by time of follow-up: (T2-T1)/follow-up models adjusted for covariates from T1
*SERPINA1* CpGs are highlighted in bold, italic. Nominally significant CpGs are in addition highlighted in grey
Significant CpGs at the nominal level, outside *SERPINA1* gene, are bolded.

Table ES7: Association of methylation at 119 CpGs in the *SERPINA** gene cluster with FEV_1_/FVC level and decline in ALSPAC children exposed to tobacco smoke (n=259)

*From *SERPINA* gene cluster located on chromosome 14 between positions: 94’641’781 and 95’235’125 (human genome build 37/hg19).
† Children and adolescents exposed to tobacco-smoke defined as: mother smoked during pregnancy and/or lived with a smoker and/or reported smoking ≥twice in their lifetime
‡ Nominal P-values, Bonferroni corrected significance level for 119 tests is P-value 4.6x10^-4^
Cross-Sectional and repeat cross-sectional models were adjusted for: study center, age, mother education, height, (height-mean(height))^2^, sex, sex*age, sex*height, and cell composition (CD8T; CD4T; NK; Bcell; Mono; Eos). Repeat cross-sectional in addition ran with a random intercept on the subject. In predictive models, annual change in lung function was computed as the difference between T2 and T1 divided by time of follow-up: (T2-T1)/follow-up models adjusted for covariates from T1
*SERPINA1* CpGs are highlighted in bold, italic. Nominally significant CpGs are in addition highlighted in grey
Significant CpGs at the nominal level, outside *SERPINA1* gene, are bolded.

Table ES8: Association of methylation at 119 CpGs in the *SERPINA** gene cluster with FEV_1_ level and decline in adult smokers from SAPALDIA, basic adjustment and adjustment for PIS and PIZ genotypes

*From *SERPINA* gene cluster located on chromosome 14 between positions: 94’641’781 and 95’235’125 (human genome build 37/hg19).
† Nominal P-values, Bonferroni corrected significance level for 119 tests is P-value 4.6x10^-4^
‡Direction of effect for SAPALDIA, ECRHS and NFBC. A positive sign indicates that an increase in methylation is associated with higher level of lung function
 (cross-sectional models) and with an attenuation of lung function decline, respectively (change model).
Repeat cross-sectional models were adjusted for: study center, age, age^2^, education, height, height^2^, sex, sex*age, (sex*age)^2^, sex*height, (sex*height)^2^, Bcell, CD4T, CD8T, Eos, Mono, NK, random intercept on the subject. In predictive models, annual change in lung function was computed as the difference between T2 and T1 divided by time of follow-up: (T2-T1)/follow-up models adjusted for covariates from T1
SERPINA1 CpGs are highlighted in bold, italic. Nominally significant CpG are in addition highlighted in grey
Significant CpGs at the nominal level, outside *SERPINA1* gene, are bolded

Table ES9: Association of methylation at 119 CpGs in the *SERPINA** gene cluster with FEV_1_/FVC level and decline in adult smokers from SAPALDIA, basic adjustment and adjustment for PIS and PIZ genotypes

*From *SERPINA* gene cluster located on chromosome 14 between positions: 94’641’781 and 95’235’125 (human genome build 37/hg19).
† Nominal P-values, Bonferroni corrected significance level for 119 tests is P-value 4.6x10^-4^
‡Direction of effect for SAPALDIA, ECRHS and NFBC. A positive sign indicates that an increase in methylation is associated with higher level of lung function
 (cross-sectional models) and with an attenuation of lung function decline, respectively (change model)
Repeat cross-sectional models were adjusted for: study center, age, age^2^, education, height, height^2^, sex, sex*age, (sex*age)^2^, sex*height, (sex*height)^2^, Bcell, CD4T, CD8T, Eos, Mono, NK, random intercept on the subject. In predictive models, annual change in lung function was computed as the difference between T2 and T1 divided by time of follow-up: (T2-T1)/follow-up models adjusted for covariates from T1
SERPINA1 CpGs are highlighted in bold, italic. Nominally significant CpG are in addition highlighted in grey
Significant CpGs at the nominal level, outside *SERPINA1* gene, are bolded
